# Essential Oil Emulsion from Caper (*Capparis spinosa* L.) Leaves: Exploration of Its Antibacterial and Antioxidant Properties for Possible Application as a Natural Food Preservative

**DOI:** 10.3390/antiox13060718

**Published:** 2024-06-13

**Authors:** Maria Merlino, Concetta Condurso, Fabrizio Cincotta, Luca Nalbone, Graziella Ziino, Antonella Verzera

**Affiliations:** Department of Veterinary Sciences, University of Messina, Polo Universitario dell’Annunziata, Viale G. Palatucci, 98168 Messina, Italy; maria.merlino@unime.it (M.M.); fabrizio.cincotta@unime.it (F.C.); luca.nalbone@unime.it (L.N.); graziella.ziino@unime.it (G.Z.); antonella.verzera@unime.it (A.V.)

**Keywords:** leaf essential oil, *Capparis spinosa* L., essential oil emulsion, volatile composition, pathogenic and spoilage foodborne bacteria, radical scavenging activity

## Abstract

This study explored, for the first time, the chemical composition and in vitro antioxidant and antibacterial activities of a caper leaf essential oil (EO) emulsion for possible food applications as a natural preservative. The EO was extracted by hydrodistillation from the leaves of *Capparis spinosa* growing wild in the Aeolian Archipelago (Sicily, Italy) and exhibited a pungent, sulphurous odour. The volatile fraction of the emulsion, analysed by SPME-GC-MS, consisted of over 100 compounds and was dominated by compounds with recognised antibacterial and antioxidant properties, namely dimethyl tetrasulfide (18.41%), dimethyl trisulfide (12.58%), methyl isothiocyanate (7.97%), and terpinen-4-ol (6.76%). The emulsion was effective against all bacterial strains tested (*Listeria monocytogenes*, *Staphylococcus aureus*, *Escherichia coli*, *Salmonella enterica* subsp. enterica serovar Enteritidis, *Pseudomonas fluorescens*), with *L. monocytogenes* exhibiting the lowest minimum inhibitory concentration (MIC = 0.02 mg/mL) while *E. coli* had the highest (MIC = 0.06 mg/mL). The emulsion had a good DPPH (2,2-diphenyl-1-picrylhydrazine) radical scavenging activity that was dose-dependent and equal to 42.98% at the 0.08 mg/mL level with an IC_50_ value of 0.099 mg/mL. Based on the results, the caper leaf EO emulsion has the potential to be proposed as a natural alternative to chemical preservatives in the food industry.

## 1. Introduction

Chemical additives are successfully used in food processing and preservation to prevent and control pathogenic and spoilage microorganisms and limit oxidation; however, their safety and impact on human health are under discussion. The increased awareness of the health risks connected with synthetic additives is driving consumer preferences towards more “green foods” and prompting the food industry to use alternative preservation methods to ensure food quality and safety [1].

In this context, essential oils extracted from various parts of plants and herbs are being extensively investigated for their potential application in the food industry [1,2,3,4,5]. Indeed, essential oils exhibit interesting biological activities, including antimicrobial, antioxidant, and anticancer properties, and are generally recognised as safe (GRAS) substances for human consumption by the Food and Drug Administration [6]. 

Caper (*Capparis spinosa* L.) is a perennial suffruticose shrub belonging to the Capparidaceae (or Capparaceae) family and the *Capparis* genus; the latter includes 142 species of tropical or subtropical origin [7]. The plant is widespread mainly in the Mediterranean basin, the Arabian Peninsula, and the Middle East, as well as in India, Malaysia, and Australia [8]. It grows both wild and as cultivated plants, preferring sunny stony soils in semi-arid coastal zones, the slopes of calcareous rocks, and the cracks of old walls. In addition, *C. spinosa* is salt tolerant and resistant to drought. Thanks to its ability to grow in harsh environments, *C. spinosa* is recommended for preventing land degradation, controlling soil erosion, and maintaining and promoting agriculture in regions subject to extreme climate changes [9]. 

This shrubby plant has a woody base and herbaceous branches with alternate simple leaves that are thick and shiny, an intense green colour, and oval-shaped. The flowers are solitary, sweetly fragrant, and showy, with white to pinkish-white petals and numerous long violet stamens. The fruits are ovate-oblong, dark green berries [8,10].

*C. spinosa* is one of the most common aromatic plants in Mediterranean cuisine. It is mainly known for its edible flower buds, named capers. The flowers, harvested in spring before they hatched, are usually processed in brine, pickled in vinegar, or preserved in granular salt, and used as a seasoning in salads, pasta, meat, sauces, and garnishes to add a tangy and briny flavour and aroma to food [8,11]. However, the whole plant is edible: the fruits, leaves, and younger shoots are also consumed salted or pickled in vinegar, and as fresh or cooked vegetables [12].

*C. spinosa* is also an important medicinal plant traditionally used to treat several health problems. Different parts of the plant (roots, bark, leaves, buds, and fruits) have been used, since ancient times, as a remedy for joint disease, haemorrhoids, rheumatism, rheumatoid arthritis, gout, fever, coughs, asthma, and inflammation [8]. In addition, anti-hypertensive, anti-hepatotoxic, anti-diabetic, anti-obesity, broncho-relaxant, anti-allergic and anti-histaminic, antibacterial, antioxidant, and anticancer effects have been recognised for caper extracts [8,13]. 

On the contrary, the composition and the biological activities of *C. spinosa* essential oil (EO) have been poorly investigated. Literature data are limited to the composition of leaf EOs from *C. spinosa* harvested in the Middle East [14,15], the composition and antioxidant activity of EOs obtained from leaves and flower buds and flower buds from Croatia [16] and Iran [17], respectively, and the composition and antimicrobial activity of the EO extracted from the aerial parts of *C. spinosa* harvested in Algeria [18] and Jordan [19].

To the best of our knowledge, the antioxidant and antimicrobial activity of caper leaf EOs from *C. spinosa* leaves has never been investigated. Therefore, considering the numerous phytochemical and pharmacological properties of *C. spinosa* and its appreciated aroma and flavour notes, especially in Mediterranean cuisine, the present study aimed to explore the potential of a caper leaf EO emulsion as a natural food preservative, through the assessment of its volatile composition, in vitro antioxidant activity, and in vitro antimicrobial effects against common food-born pathogenic and spoilage bacteria. The essential oil was extracted through the hydrodistillation of leaves of *C. spinosa* harvested in the Aeolian Archipelago, one of the main production areas in Italy, and formulated as an oil-in-water emulsion for preventing volatilisation and loss and increasing the oil dispersion capacity. 

## 2. Materials and Methods

### 2.1. Plant Material

Leaves of *C. spinosa* were harvested from wild-grown caper plants in the northwest region of the Island of Lipari (Aeolian Archipelago, Sicily, Italy) in the first half of June 2023.

The collected leaves were quickly transported to the laboratory, washed under cold running water, and dried with a cloth. Finally, the leaves were dried with hot air at 50 °C at a constant air velocity (1.5 m/s) until a moisture loss of 95–96% was reached (~6 h) and ground into powder.

The taxonomic identification of the plant was carried out by Dr. Fabio Mondello at the Department of Chemical, Biological, Pharmaceutical, and Environmental Sciences of Messina University, and a voucher specimen was deposited in the same department.

### 2.2. Essential Oil Extraction

Approximately 200 g of powdered dried caper leaves was mixed with 1.0 L of distilled water and subjected to hydrodistillation for 3 h. The oil was dried overnight over anhydrous sodium sulphate; packaged in sealed, amber-coloured glass vials; and stored at room temperature in a dark, cool, and dry place until use. The extraction procedure was performed in triplicate.

### 2.3. Emulsion Preparation

An oil-in-water emulsion was prepared by mixing 40 mg of caper leaf EO and 500 mL of distilled water (conc. 0.08 mg/mL). The mixture was homogenised at 12,000 rpm for 2 min using an Ultra-Turrax T18 homogeniser (Janke & Kunkel, IKA Instruments, Staufen, Germany). The prepared emulsion was stable until use. All the analyses on the oil-in-water emulsion were performed in triplicate.

### 2.4. SPME-GC-MS Analysis

The volatile compounds of the caper leaf EO emulsion were extracted using the solid-phase microextraction technique (SPME) using a DVB/CAR/PDMS fibre with a 50/30 μm film thickness (Supelco, Bellefonte, PA, USA). For the extraction, an aliquot of 18 mL of the EO emulsion plus 6 g of NaCl (Sigma-Aldrich, Milan, Italy) were placed in a 40 mL vial sealed with a ‘mininert’ valve (Supelco, Bellefonte, PA, USA). The sample was thermally equilibrated at 35 °C for 20 min in a water bath and, subsequently, the SPME fibre was exposed to the sample headspace for 20 min under magnetic stirring.

The extracted volatiles were analysed by a Shimadzu GC 2010 Plus gas chromatograph coupled with a TQMS 8040 triple-quadrupole mass spectrometer (Shimadzu, Milan, Italy). Two capillary columns with different polarities were used: (1) a VF-WAXms, 60 m, 0.25 mm i.d., 0.25 μm film thickness polar column (Agilent Technologies Italia S.p.A.; Milan, Italy) and (2) a DB-5 ms, 30 m, 0.25 mm i.d., 0.25 μm film thickness apolar column (Agilent Technologies Italia S.p.A.; Milan, Italy). The conditions were as follows: injection mode, splitless; oven temperature, (1) 50 °C for 5 min, up to 190 °C at 3 °C/min and to 240 °C at 6 °C/min for the polar column, (2) 45 °C for 5 min, increased to 110 °C at 4 °C/min, to 240 °C at 2 °C/min, and to 260 °C at 20 °C/min, held at 260 °C for 2 min for the apolar column; carrier gas, helium at a constant flow of 1 mL/min; transfer line temperature, 250 °C; acquisition range, 40 to 400 m/z; and scan speed, 1250. The identification of the volatiles was performed using the mass spectral data, the NIST’ 14 (NIST/EPA/NIH Mass Spectra Library, version 2.0, Gaithersburg, MD, USA), and FFNSC 3.0 database (Shimadzu, Kyoto, Japan), linear retention indices (LRIs) calculated according to the Van Den Dool and Kratz equation, and injection of available standards [20]. Quantitative results were expressed as a percentage of the total peak area.

### 2.5. Antibacterial Activity

The antimicrobial activity of the caper leaf EO emulsion was tested against different ATCC bacterial strains, namely *Listeria monocytogenes* 7644, *Staphylococcus aureus* 25923, *Escherichia coli* 25922, *Salmonella enterica* subsp. *enterica* serovar Enteritidis 13076, and *Pseudomonas fluorescens* 13525. All the strains were stored at –80 °C at the microbial collection of the “Food Microbiology Laboratory” of the Department of Veterinary Sciences, University of Messina (Messina, Italy). The tested strains were prepared by plating a loopful of the frozen stock onto nutrient agar plates (Biolife, Milan, Italy) and incubating overnight at 37 °C before each analysis.

The minimum inhibitory concentration (MIC) and minimum bactericidal concentration (MBC) were assessed for each of the strains. The MIC was evaluated using the broth microdilution method according to Kowalska-Krochmal and Dudek-Wicher [21] using the o/w emulsion prepared as described above. The o/w emulsion was diluted using brain heart infusion broth (BHI) (Biolife, Milan, Italy) to final concentrations of 0.06, 0.04, 0.02, and 0.01 mg/mL. Several U Eppendorf tubes (Biosigma, Cona, Italy; 2 mL volume) were arranged in rows on racks and filled with 1 mL of caper leaf EO emulsion at different concentrations. A fresh inoculum of each bacterium was grown in BHI at 37 °C for 24 h. Subsequently, a microbial suspension from the broth cultures was inoculated in each U Eppendorf tube obtaining a final concentration of ~10^4^ CFU/mL. Then, the U Eppendorf tubes were incubated at 37 °C for 24 h. The positive control consisted of broth medium inoculated with microbial suspensions while the uninoculated broth medium with the highest concentration of each compound served as the negative control. The lowest concentration of each compound in which there was no visible growth (turbidity of the broth medium) was determined to be the MIC. 

The MBC was determined by inoculating the suspensions from each U Eppendorf on nutrient agar plates (Biolife, Milan, Italy) and incubating them at 37 °C for 24 h. The lowest concentration in which there was no microbial growth (no colonies in the culture medium) was determined to be the MBC.

The MIC and MBC analyses were performed in triplicate.

### 2.6. Antioxidant Activity

The antioxidant activity of the caper leaf EO emulsion was assessed by measuring the free radical scavenging ability towards the DPPH (2,2-diphenyl-1-picrylhydrazine) radical (Merk Life Science S.r.l., Milan, Italy).

For the DPPH assay, 2000 μL of a DPPH methanolic solution (0.149 mM) was added to 500 μL of the EO emulsion and incubated at room temperature for 1 h. Similarly, a reference sample was prepared using 500 μL of solvent (distilled water) as a substitution for the EO emulsion. The absorbance of the mixtures was read at 517 nm using a double-beam UV–Vis spectrophotometer (UV-2550, Shimadzu, Milan, Italy) against a 4:1 *v*/*v* MeOH/H_2_O mixture used as a blank. The EO emulsion was tested in a concentration range of 0.016 to 0.080 mg/mL. Additionally, butylated hydroxytoluene (BHT) methanolic solutions (Merk Life Science S.r.l., Milan, Italy) were tested under the same experimental conditions and in the same concentration range, and they were utilised as positive controls. 

The percentage radical scavenging activity [*RSA*(%)] of the caper leaf EO emulsion, as well as BHT, was calculated using the following equation:

$$RSA\;\left(\%\right)=\left(\frac{Ar-As}{Ar}\right)\times 100$$

where *Ar* is the absorbance at 517 nm of the reference sample, and *As* is the absorbance at 517 nm of the samples containing the EO emulsion or BHT solution. 

The concentration required to scavenge 50% of the initial DPPH radical (IC_50_) was determined by plotting the scavenging percentage against the test sample concentration (mg/mL); a linear correlation was found in both the EO emulsion (y=540.85 x, R^2^ = 0.999) and BHT solution (y=1015.3 x, R^2^ = 0.995).

### 2.7. Statistical Analysis

Excel 2010 software (Microsoft, Milan, Italy) was used to calculate the means and standard deviations of the three replicates. To determine significant differences, the means were analysed by one-way ANOVA and Duncan’s multiple range test at a confidence level of 95% or more, using the Microsoft XLstat software 2014 (Addinsoft, Paris, France).

## 3. Results and Discussion

The hydrodistillation extraction of caper leaves allowed us to obtain a yellowish oil with a pungent sulphurous smell and a specific gravity of 1.1308 ± 0.0131 g/mL, consistent with the findings of Afsharypuor et al. [15]. The extraction yield was 0.021 ± 0.001%; this is a quite low yield but in line with the data reported in the literature when this extraction procedure was applied to caper leaves [15,18].

The antimicrobial and antioxidant activities of the caper leaf EO was tested using an oil-in-water emulsion. EO emulsification offers some advantages for food applications, such as a decrease in viscosity, an increase in dispersion capacity, and the prevention of EO volatilisation and loss [22,23]. Instead, the effect of emulsification on food microorganisms is still controversial; some studies verified that emulsification increases the antimicrobial effects of EOs against food microorganisms by increasing their ability to damage cell membranes [24], while others proved that the EO antimicrobial activity depends on its chemical composition rather than droplet size [22].

### 3.1. Volatile Fraction Composition

The oil-in-water emulsion was chemically characterised by HS-SPME-GC-MS: 34 terpenes, 13 C_13_-norisoprenoids, 10 sulphur and 2 nitrogen compounds, 13 ketones, 15 alcohols, 7 aldehydes, 6 esters, and 4 furanic compounds were identified, for a total of over 100 compounds. The percentage composition of the volatile fraction of the caper leaf EO emulsion are reported in Table 1. The most quantitatively represented classes of compounds were terpenoids, sulphur compounds, and C_13_-norisoprenoids, which together accounted for more than 86% of the total volatile fraction. 

The class of terpenoids was represented almost exclusively by oxygenated monoterpenes, with terpinen-4-ol, linalool, eucalyptol, thymol, α-terpineol, and menthone as the main compounds. Caryophyllene oxide and α-bisabolol oxide B were the only sesquiterpenes here identified, although they occurred at low percentages. 

Among the sulphur compounds, isothiocyanates, methyl polysulfides, and cyclic octatonic sulphur were found, the latter being already reported as a constituent of pickled capers [25]. Isothiocyanates arise from the enzymatic hydrolysis of glucosinolates by the action of myrosinase. Glucosinolates are anionic secondary metabolites principally found in the plant order Brassicales, which includes the large families of Brassicaceae and Capparaceae [26]. Bianco et al. [27] reported glucocapparin and glucobrassicin as the main glucosinolates in *C. spinosa* from Southern Italy; from glucocapparin hydrolysis, methyl isothiocyanate arises, whereas from glucobrassicin hydrolysis, a highly unstable isothiocyanate occurs which rapidly degrades into indole-3-methanol and thiocyanate ions [28]. Methyl isothiocyanate and indole-3-methanol were present in the caper leaf EO emulsion at the highest percentages among the glucosinolate derivatives; indeed, ethyl isothiocyanate, isopropyl isothiocyanate, butyl isothiocyanate, and isobutyl isothiocyanate were present at levels below 0.1%. 

Dimethyl disulfides, dimethyl trisulfides, dimethyl tetrasulfides, and dimethyl pentasulfides were among the main constituents of the volatile profile of our sample. Dimethyl polysulfides originate from methiin, namely (+)-S-methyl-L-cysteine sulfoxide, by the action of cysteine sulfoxidelyase. Methiin has been reported as the only S-alk(en)yl-L-cysteine sulfoxide compound in plants of the Brassicaceae family; its level can differ greatly among the genera of the family and, in the case of cruciferous vegetables, it is much higher than that of glucosinolates [29,30]. To the best of our knowledge, there are no studies focused on methiin occurrence and levels in Capparaceae plants even if dimethyl sulfide and dimethyl polysulfides have been reported among the volatile compounds of caper buds [11,25,31]. Very recent studies reported the formation of dimethyl disulfide and dimethyl trisulfide through thermal degradation of glucoerucin in rapeseed oil at 150 °C [32]; however, concerning our sample, this route for polysulfide formation seems unlikely since the leaves were not subjected to such high temperatures. 

Dihydroedulan I and (*E*)-β-ionone quantitatively prevailed among the C_13_-norisoprenoids identified in the caper leaf EO emulsion. C_13_-norisoprenoids are 13-carbon skeleton compounds arising from carotenoid breakdown through enzymatic (by the action of carotenoid cleavage dioxygenases) and/or non-enzymatic (photo-oxygenation, thermal degradation in aqueous medium, and acid hydrolysis of intermediate megastigma precursors) pathways. C_13_-norisoprenoids are common plant volatile compounds, present in leaves, flowers, and fruits. They have pleasant floral and fruity notes and, thanks to their very low odour thresholds (at ppt level, in water), strongly contribute to the aroma of flowers and fruits. In particular, C_13_-norisoprenoids are the most important aroma compounds in some grape varieties and their wines, such as Cabernet Sauvignon, Merlot, Syrah, and Chardonnay [33]. Dihydroedulan I, probably biosynthesised from α-ionone, has a camphoraceous aroma and it is responsible for the characteristic odour of elderberry juice where it occurs at relatively high concentrations; moreover, it has also been detected in the pulp and juice of purple passion fruit [34,35]. β-ionone is formed directly from the enzymatic and/or non-enzymatic degradation of β-carotene [36], and it is responsible for violet and woody notes. Moreover, recent studies have highlighted its anti-inflammatory and cancer-preventing activities and the related benefits for human health [37]. 

Data on the chemical composition of caper leaf EO are currently scarce and only refer to the EO from *C. spinosa* grown in the Middle East [14,15]. In all cases, only a few compounds, i.e., less than fifteen, were reported; thymol, octanoic acid, methyl isothiocyanate, and 2-hexenal were the major constituents of the EO from leaves of *C. spinosa* grown in Syria [14], whereas thymol, isopropyl isothiocyanate, 2-hexenal, and butyl isothiocyanate prevailed in the leaf EO of *C. spinosa* var. *mucronifolia* from Iran [15]. In addition, the EO composition of leaves and flower buds of *C. spinosa* from Croatia was investigated by Kulisic-Bilusic et al. [16], identifying 10 compounds overall, with methyl isothiocyanate at a level over 90%. A greater number of compounds were instead reported in the EO extracted from the plant’s aerial parts: 29 components were reported by Muhaidat et al. [19] in the EO of *C. spinosa* aerial parts harvested in the arid bottoms of the Dead Sea valley (Jordan), among which, isopropyl isothiocyanate, methyl isothiocyanate, butyl isothiocyanate, 3-p-menthene, 2-butenyl isothiocyanate, and 3-methylthio-1-hexanol prevailed; later Benachour et al. [18] identified 33 compounds, mainly palmitic acid, nonanal, 2,5-dimethoxy-p-cymene, and octacosane, in the aerial part EO of *C. spinosa* grown in Algeria.

This is the first report that fully characterised the EO extracted by hydrodistillation from the leaves of *C. spinosa*, identifying more than 100 compounds, most of which are reported for the first time in the EO of caper leaves. 

### 3.2. Antibacterial Activity

The antibacterial activity of the caper leaf EO emulsion was tested against *L. monocytogenes* ATCC 7644, *S. aureus* ATCC 25923, *S.* Enteritidis ATCC 13076, *E. coli* ATCC 25922, and *P. fluorescens* ATCC 13525. The MIC and MBC values were the same in all three replicates performed for each strain tested. 

The emulsion showed inhibitory effects against all the strains tested (both Gram-positive and Gram-negative strains (Table 2, Figure S1), and revealed an efficacy against the foodborne pathogens *S.* Enteritidis and *L. monocytogenes* which were, respectively, the second and fifth most reported foodborne zoonotic agents in Europe in 2022 [38]. In particular, *L. monocytogenes* was the most sensitive among the tested strains with an MIC value of 0.02 mg/mL, whereas *E. coli* was the less sensitive (MIC value equal to 0.06 mg/mL). Antimicrobial activity was also detected against *P. fluorescens*, one of the most common spoilage agents in refrigerated foods [39]. 

The antibacterial activity of the caper leaf EO emulsion was consistent with its chemical composition as many of the identified compounds have proven antibacterial activity. Ak(en)yl polysulfides display an antimicrobial activity that increases as the number of sulphur atoms increases and the number of carbon atoms in the alk(en)yl group decreases [40]. Isothiocyanates also exert antimicrobial activity against both pathogenic and food spoilage bacteria, with methyl isothiocyanate able to inhibit *Salmonella* spp., *E. coli*, and *L. monocytogenes* [41]. Thus, we can suppose that dimethyl trisulfide, dimethyl tetrasulfide, dimethyl pentasulfide, and methyl isothiocyanate, which are among the most abundant compounds of caper leaf essential oil, as well as the main oxygenated monoterpenes linalool, α-terpineol, terpinen-4-ol, thymol, and eucalyptol made a marked contribution to the observed antimicrobial activity. Linalool, α-terpineol, terpinen-4-ol, and thymol have broad-spectrum antibacterial activity and were found to be effective against, among others, *S. aureus*, *E. coli*, *S. enterica*, and *L. monocytogenes* [42,43]. Regarding eucalyptol, little information is present in the literature on the antimicrobial activity of its pure compounds; however, studies on eucalyptus EO (eucalyptol ≥ 60%) revealed antibacterial activity against Gram-positive and Gram-negative bacteria that are resistant to commonly used antimicrobial agents [44]. 

Although several studies are available in the literature on the antimicrobial activity of *C. spinosa* extracts, only a few have investigated the antibacterial effects of its essential oils, which exclusively focused on the essential oil extracted from the aerial part of the plant [18,19]. Instead, the antibacterial activity of caper leaf EO has never been explored before. Muhaidat et al. [19] tested the antibacterial activity of EO from the aerial parts of *C. spinosa* harvested in Jordan against different Gram-positive and Gram-negative ATCC strains; a greater inhibitory efficacy was observed against Gram-positive bacteria (*Staphylococcus aureus*, *Staphylococcus epidermidis*, *Streptococcus faecalis*, *Bacillus cereus*, and *Micrococcus luteus*) compared to Gram-negative bacteria (*Salmonella* Typhimurium, *Pseudomonas aeruginosa*, *Klebsiella pneumoniae*, and *Enterobacter aerogenes*), with the lowest activity against *E. coli*. More recently, Benachour et al. [18] investigated the antibacterial activity of the essential oil from the aerial parts of *C. spinosa* collected in Algeria they reported antibacterial effects against the ATCC strains of *S. aureus*, *Klebsiella pneumoniae*, *B. cereus*, *P. aeruginosa*, *S. enterica*, *Proteus mirabilis*, and *Enterococcus faecalis*, whereas no activity was detected against *E. coli*. 

These two studies revealed a low antibacterial efficacy of the caper aerial part EO against *E. coli* in contrast with the inhibitory effects against *E. coli* exhibited herein by the leaf EO emulsion. Indeed, the EOs from caper aerial parts tested in the above-cited studies had a chemical composition that is extremely different from that of our caper leaf EO emulsion, lacking compounds with proven antimicrobial activity, such as sulphur compounds and monoterpene alcohols.

Considering the results obtained herein, we speculate that the use of caper leaf EO emulsions in foods could improve their quality and safety. On the one hand, the effectiveness against spoilage bacteria could be exploited to extend foods’ shelf life. On the other hand, they could be evaluated as a natural substitute for common additives that fight foodborne pathogens. The effectiveness against *P. fluorescens* would suggest the use of the caper leaf EO emulsion in some foods such as fishery products in which this psychrotrophic bacterium represents one of the main spoilage agents. In this regard, Shafaghi Rad and Nouri [17] added *C. spinosa* fruit essential oil in fish burgers and reported inhibitory effects against the psychrophilic flora that, after 8 days of refrigerated storage, was 6 log CFU/g lower than in untreated control burgers. In addition, the antimicrobial activity against *L. monocytogenes* suggests its use in ready-to-eat foods, such as some meat or fishery products, where the use of caper leaf essential oil can also add an appreciable pungent and sulphureous flavour to the product. Ready-to-eat foods are those most at risk for *L. monocytogenes* contamination [34] and the essential oil activity could, on the one hand, inhibit the growth of the microorganism and, on the other hand, act as a flavouring agent. Furthermore, the emulsification makes the direct use of the EO more likely in foods, which is different from other more studied extracts such as ethanolic ones whose use is affected by the alcohol content which could influence consumer acceptance and perception.

However, since the in vitro results may not reflect the antibacterial activity in a much more complex matrix such as food—due to the influence of factors such as pH, water activity, and interactions with food components—the EO emulsion applications in different food matrices are under investigation.

### 3.3. Antioxidant Activity

**Figure 1:**
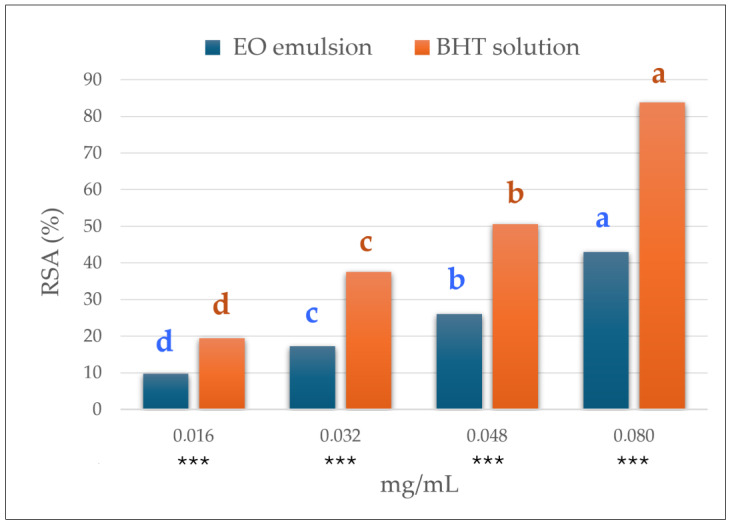
Antioxidant activity (DPPH assay) of caper leaf EO emulsion and BHT solution. Different letters indicate statistically significant differences among concentrations according to Duncan’s multiple range test at *p* < 0.001. *** = statistically significant differences between EO emulsion and BHT solution at *p* < 0.001.

Concerning the primary antioxidant capacity, specifically the ability to scavenge free radicals, the caper leaf EO emulsion exhibited dose-dependent DPPH radical inhibition, equal to 42.98 ± 0.02% at the 0.08 mg/mL level (Figure 1) and an of 0.099 mg/mL which revealed a lower scavenging activity than the BHT standard (IC_50_ 0.049 mg/mL). However, 0.099 mg/mL could be considered a very good IC_50_ value considering that, according to extraction yield, an amount of 0.5 g of caper leaves was enough to obtain 0.105 mg of EO. In addition, the values of the percentage of DPPH inhibition and IC_50_ obtained for the caper leaf EO emulsion were higher than those reported in the literature for essential oils from both caper and other plants, suggesting a good antioxidant capacity of the caper leaf EO emulsion compared to other essential oils. In particular, Kulisic-Bilusic et al. [16] described DPPH inhibitory percentages of 7.55% and 10.45% at the concentrations of 0.2 g/L and 2 g/L, respectively, for the EOs of caper floral buds and leaves. The leaf EO of *Capparis ovata* Desf. var. *canescens* cultivated in Turkey inhibited 73.5% of DPPH radicals at the 500 μg/mL level [45]. Additionally, Anthony et al. [46] analysed 423 essential oils of 48 different botanical families and reported IC_50_ values ≤ 100 μg/mL for only 10 of them. The scavenging activity of the caper leaf EO suggests a possible use as an antioxidant agent for fresh and processed meat and meat products. In these foodstuffs, the oxidation process proceeds through a free radical autocatalytic mechanism involving unsaturated fatty acids, and it leads to lipid degradation, development of rancidity flavours, and changes in meat colour. The lipid oxidation process is the main factor responsible for quality loss during meats’ shelf life and consumer rejection; thus, new solutions for controlling or minimising this process are always of great interest to the meat industry [47]. 

The antioxidant activity of the caper leaf EO can be related to its terpenoid profile. It is well known that, thanks to their antioxidant behaviour, terpenes provide protection against oxidative stress in ageing processes and several diseases, like cancer, diabetes, and neurodegenerative and cardiovascular diseases [48]. The antioxidant properties of terpenoids are dependent on their chemical structure and functional group, for example, (1) monoterpenes have better radical scavenging properties than sesquiterpenes; (2) among monoterpenes, hydrocarbons have the highest activity, whereas oxygenated monoterpenes’ scavenging activity follows the decreasing order of alcohols > aldehydes and ketones; (3) the oxygenated sesquiterpenes exhibit good scavenging properties, similar to those of oxygenated monoterpenes; and (4) sesquiterpenes hydrocarbons have very low radical scavenging properties [48]. The terpenoid profile of the caper leaf EO emulsion was constituted almost entirely by oxygenated monoterpenes, of which, about 60% was alcohols. The most quantitatively represented terpenes were terpinen-4-ol (7.13%), linalool (5.03%), eucalyptol (3.27%), α-terpineol (1.91%), menthone (1.71%), and 1,5,9,9-tetramethyl-2-methylene-spiro[3.5]non-5-ene (1.73%); the latter was the only hydrocarbon present in the terpene fraction of the caper leaf EO emulsion. The scarce presence of hydrocarbon monoterpenes can account for the moderate antioxidant activity of the emulsion with respect to BHT. The antioxidant activity of isothiocyanates and organopolysulfides is also well known [49,50], although the compounds identified herein have never been tested for their antioxidant activity as pure compounds.

Therefore, it is possible to suppose that both the main monoterpenes and sulphur compounds were responsible for the antioxidant activity exhibited by the caper leaf EO emulsion as well as its antibacterial effects that were discussed in the previous section.

## 4. Conclusions

The results of this study revealed that an oil-in-water emulsion of leaf EO extracted by hydrodistillation from *C. spinosa* harvested in the Aeolian Archipelago had a very complex volatile profile, consisting of over 100 compounds and dominated by compounds with recognised antibacterial and antioxidant properties, such as methyl isothiocyanate, dimethyl polysulfides, and oxygenated monoterpenes. Indeed, the leaf EO exhibited a good antioxidant capacity (IC_50_ 0.099 mg/mL) and was effective against all the tested bacterial strains at low concentrations (≤0.06 mg/mL), especially against *L. monocytogenes*.

This is the first study to deeply characterise the leaf EO of *C. spinosa*, identifying a large number of chemical compounds, most of which were described for the first time in the EO of caper leaves; the study also correlated the EO composition with its in vitro antibacterial and antioxidant capacities. The current study demonstrated that caper leaf essential oil in the form of an oil-in-water emulsion has the potential to be proposed as a natural alternative to chemical preservatives and other synthetic antioxidants in the food industry. Despite the promising results, this study is only preliminary and further investigations are ongoing to comprehensively explore the antimicrobial and antioxidant activities of the EO emulsion in food matrices.

## Figures and Tables

**Table 1:** Composition of volatile constituents and classes of substances of essential oil (EO) emulsion from caper (*Capparis spinosa* L.) leaves.

Compounds	LRI ^1^ on DB-5	LRI ^1^ on VF-WAX	Amount ^2^ (% ± SD)
**Sulphur-containing compounds**			
Methyl isothiocyanate	722	1243	7.97 ± 0.30
Dimethyl disulfide	725	1080	1.25 ± 0.08
Ethyl isothiocyanate	801	-	0.05 ± 0.00
Isopropyl isothiocyanate	827	1179	0.09 ± 0.00
Butyl isothiocyanate	928	1312	0.09 ± 0.00
Isobutyl isothiocyanate	951	1336	0.05 ± 0.00
Dimethyl trisulfide	969	1380	12.58 ± 0.93
Dimethyl tetrasulfide	1218	1750	18.41 ± 1.01
Dimethyl pentasulfide	1454	2185	4.28 ± 0.39
Cyclic octatonic sulphur	2014	-	0.57 ± 0.01
**All**			**45.33 ± 2.09**
**Terpenenoids**			
Eucalyptol	1032	1209	3.10 ± 0.10
Linalool	1102	1541	4.77 ± 0.18
Grandlure III	1111	1799	0.04 ± 0.00
Sabinol	1143	-	0.36 ± 0.02
(*E*)-*p*-Mentha-2,8-dien-1-ol	1123	1641	0.11 ± 0.01
Limona ketone	1132	-	0.05 ± 0.00
Dihydrolinalool	1137	1449	0.07 ± 0.00
(*Z*)-*p*-Mentha-2,8-dien-1-ol	1139	1656	0.07 ± 0.00
Camphor	1147	1498	0.27 ± 0.02
1,4-Dimethyl-4-acetylcyclohexene	1150	1504	0.13 ± 0.01
Karahanaenone	1153	-	0.06 ± 0.00
Menthone	1156	1472	1.63 ± 0.09
Pinocarvone	1163	1566	0.06 ± 0.00
Isomenthone	1166	1476	0.57 ± 0.02
Neomenthol	1171	1574	0.80 ± 0.03
Terpinen-4-ol	1182	1591	6.76 ± 0.32
*p*-Cymen-8-ol	1190	1850	0.55 ± 0.02
Methyl salicylate	1191	1745	0.12 ± 0.01
α-Terpineol	1198	1695	1.82 ± 0.05
Nerol	1225	1770	0.12 ± 0.01
(*Z*)-Carveol	1232	1846	0.09 ± 0.00
Pulegone	1237	1637	0.14 ± 0.01
Carvone	1243	1739	0.26 ± 0.01
Geraniol	1251	1860	0.49 ± 0.02
β-Cyclohomocitral	1253	1598	0.15 ± 0.01
Perilla alcohol	1301	2021	0.62 ± 0.03
Thymol	1307	2154	2.03 ± 0.08
4-Vinyl-guaiacol	1310	2203	0.90 ± 0.03
1,5,9,9-Tetramethyl-2-methylene-spiro[3.5]non-5-ene	1329	-	1.64 ± 0.08
Eugenol	1350	2172	0.49 ± 0.02
Methyl-eugenol	1400	2011	0.12 ± 0.00
Caryophyllene oxide	1576	1990	0.14 ± 0.01
α-Bisabolol oxide B	1650	-	0.23 ± 0.02
Phytone	1840	2118	0.06 ± 0.00
**All**			**28.82 ± 0.72**
**C13-Norisoprenoids**			
2(1H)-Naphthalenone, 3,4,4a,5,6,7-hexahydro-1,1,4a-trimethyl	1277	-	0.25 ± 0.01
Dihydroedulan I	1287	1542	2.06 ± 0.09
(*Z*)-Theaspirane	1297	1561	0.24 ± 0.02
(*E*)-Theaspirane	1313	-	0.91 ± 0.04
Dihydroedulan II	1325	1513	0.60 ± 0.02
6-Methyl-5-(1-methylethylidene)-6,8-nonadien-2-one isomer I	1355	-	1.57 ± 0.01
(*E*)-β-Damascenone	1377	-	0.58 ± 0.01
6-Methyl-5-(1-methylethylidene)-6,8-nonadien-2-one isomer II	1389	-	1.02 ± 0.02
(*E*)-β-Damascone	1407	1782	0.10 ± 0.00
3,4-Didehydro-7,8-dihydro-β-ionone	1413	-	0.41 ± 0.00
(*E*)-α-Ionone	1421	1550	0.31 ± 0.00
7,8-Epoxy-α-ionone	1433	-	1.74 ± 0.00
(*E*)-β-Ionone	1478	1928	2.33 ± 0.12
**All**			**12.12 ± 0.15**
**Nitrogen-containing compounds**			
1H-Indole	1292	2435	0.96 ± 0.00
1H-Indole-3-methanol	1383	-	3.03 ± 0.18
**All**			**3.99 ± 0.10**
**Alcohols**			
(*Z*)-3-Hexen-1-ol	860	1384	0.04 ± 0.00
1-Hexanol	876	1357	0.03 ± 0.00
2,4-Dimethyl-3-heptanol	946	-	0.01 ± 0.00
1-Heptanol	979	1457	0.09 ± 0.00
1-Octen-3-ol	984	1448	0.22 ± 0.02
6-Methyl-5-hepten-2-ol	998	1468	0.06 ± 0.00
3-Ethyl-hexanol	1043	1483	0.13 ± 0.01
2-Octen-1-ol	1062	1637	0.30 ± 0.02
1-Nonen-4-ol	1109	-	0.76 ± 0.03
1-Octanol	1076	1557	1.05 ± 0.09
1-Nonanol	1176	1657	0.67 ± 0.03
1-Decanol	1273	1758	0.67 ± 0.03
1-Dodecanol	1475	1958	0.65 ± 0.03
1-Tetradecanol	1677	2159	0.17 ± 0.01
1-Hexadecanol	1880	2359	0.08 ± 0.00
**All**			**4.91 ± 0.18**
**Aldehydes**			
(*E*)-2-Hexenal	856	1222	0.02 ± 0.00
Octanal	1005	1284	0.08 ± 0.00
Nonanal	1105	1384	0.40 ± 0.02
Decanal	1208	1486	0.34 ± 0.02
2-Phenyl-2-butenal	1266	1922	0.18 ± 0.01
4-Methyl-2-phenyl-2-pentenal	1364	1932	0.11 ± 0.00
5-Methyl-2-phenyl-2-hexenal	1482	2083	0.26 ± 0.01
**All**			**1.40 ± 0.02**
**Esters**			
2-Propenyl hexanoate	1088	1370	0.10 ± 0.00
Methyl 2,6-cresoate	1311	1970	0.70 ± 0.03
Massoia lactone	1471	2241	0.11 ± 0.00
(*Z*)-3-Hexenyl benzoate	1567	2093	0.50 ± 0.02
Isopropyl tetradecanoate	1824	2041	0.11 ± 0.01
2-Phenylethyl benzoate	1845	2658	0.04 ± 0.00
**All**			**1.56 ± 0.03**
**Ketones**			
3-Heptanone	888	1156	0.01 ± 0.00
2-Heptanone	893	1187	0.04 ± 0.00
3-Methyl-2-cyclohexen-1-one	1059	1592	0.06 ± 0.00
1-Octen-3-one	979	1307	0.27 ± 0.02
6-Methyl-5-hepten-2-one	988	1342	0.11 ± 0.01
2-Octanone	993	1289	0.01 ± 0.00
Acetophenone	1066	1656	0.02 ± 0.00
2-Nonanone	1093	1389	0.03 ± 0.00
(*E*,*E*)-3,5-Octadien-2-one,	1097	1521	0.04 ± 0.00
6-Methyl-3,5-heptadien-2-one	1108	1582	0.04 ± 0.00
2-Nonen-4-one	1127	1470	0.43 ± 0.02
3-Nonen-2-one	1142	1518	0.10 ± 0.00
Benzophenone	1621	2427	0.17 ± 0.01
**All**			**1.34 ± 0.03**
**Furanoic compounds**			
Furfural	831	1467	0.01 ± 0.00
2,5-Diethyl-tetrahydrofuran isomer I	889	-	0.13 ± 0.01
2,5-Diethyl-tetrahydrofuran isomer II	898	-	0.16 ± 0.01
6-Methyl-6-(5-methylfuran-2-yl) heptan-2-one	1480	-	0.24 ± 0.01
**All**			**0.54± 0.01**

^1^ Linear retention indexes calculated according to the Van Den Dool and Kratz equation; ^2^ percentage of peak area in TIC chromatogram (mean ± standard deviation of 3 replicates).

**Table 2:** Minimum inhibitory concentrations (MICs) and minimum bactericidal concentrations (MBCs) of EO emulsion from caper (*Capparis spinosa* L.) leaves against different ATCC bacterial strains.

ATCC Strain	MIC (mg/mL)				MBC (mg/mL)			
	0.06	0.04	0.02	0.01	0.06	0.04	0.02	0.01
*L. monocytogenes* 7644	NG ^1^	NG	NG	G	NG	G ^2^	G	G
*S. aureus* 25923	NG	NG	G	G	NG	G	G	G
*E. coli* 25922	NG	G	G	G	NG	G	G	G
*S.* Enteritidis 13076	NG	NG	G	G	NG	G	G	G
*P. fluorescens* 13525	NG	NG	G	G	NG	G	G	G

^1^ NG = no growth. ^2^ G = growth.

## Data Availability

Data is contained within the article and Supplementary Materials.

## Supplementary Materials

The following supporting information can be downloaded at: https://www.mdpi.com/article/10.3390/antiox13060718/s1.
