# Supplementary figures and images for: Essential Oil Emulsion from Caper (Capparis spinosa L.) Leaves: Exploration of Its Antibacterial and Antioxidant Properties for Possible Application as a Natural Food Preservative
Source: Antioxidants (Basel). 2024 Jun 13;13(6):718. doi: 10.3390/antiox13060718 (PMC11200896; doi:10.3390/antiox13060718)

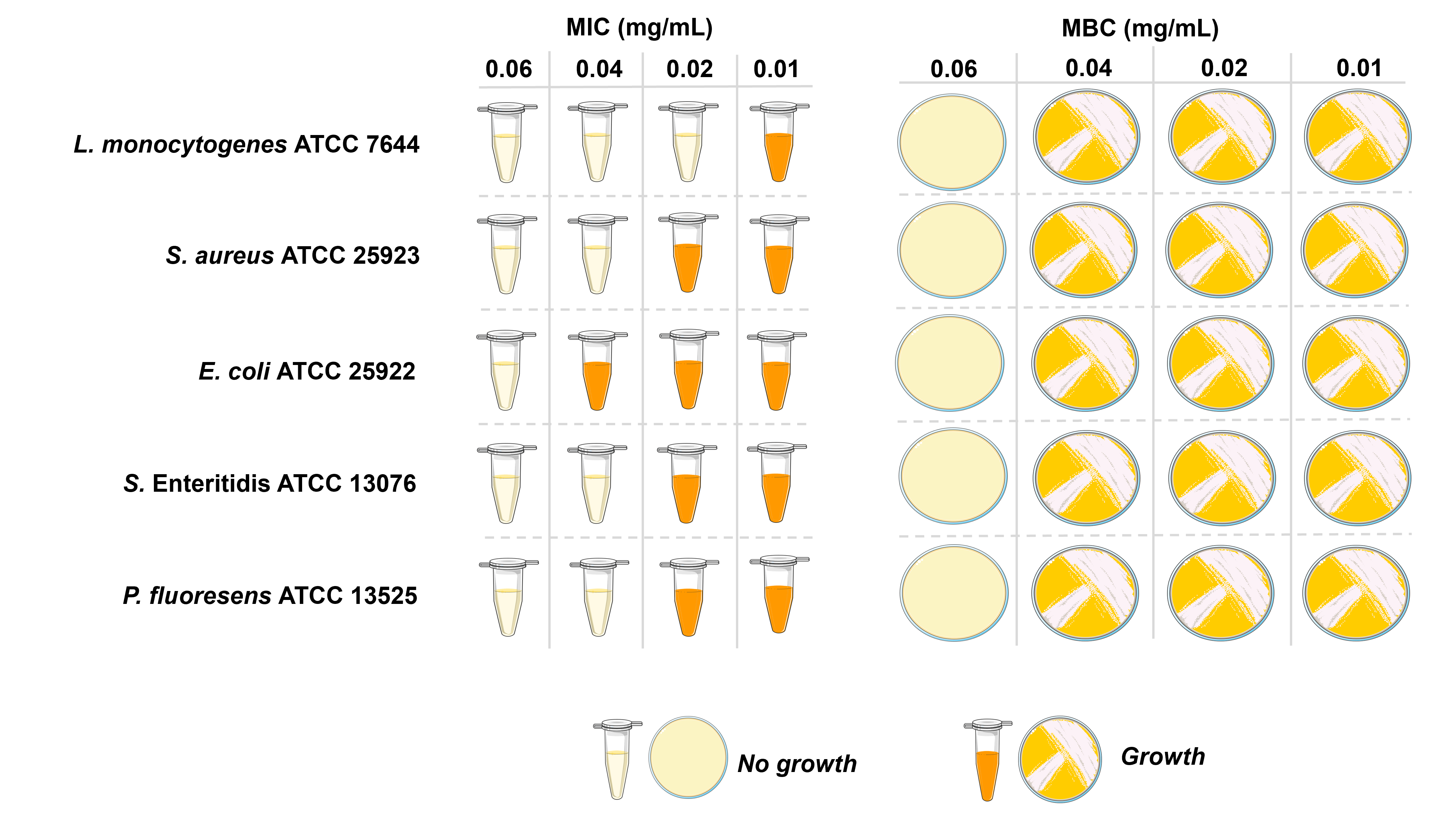

Supplement: Supplementary file 1 [file antioxidants-13-00718-s001.zip › antioxidants-3037674-supplementary.tif]
